# Genetic Deletion of TREK-1 or TWIK-1/TREK-1 Potassium Channels does not Alter the Basic Electrophysiological Properties of Mature Hippocampal Astrocytes *In Situ*

**DOI:** 10.3389/fncel.2016.00013

**Published:** 2016-02-03

**Authors:** Yixing Du, Conrad M. Kiyoshi, Qi Wang, Wei Wang, Baofeng Ma, Catherine C. Alford, Shiying Zhong, Qi Wan, Haijun Chen, Eric E. Lloyd, Robert M. Jr. Bryan, Min Zhou

**Affiliations:** ^1^Department of Neuroscience, The Ohio State University Wexner Medical CenterColumbus, OH, USA; ^2^Department of Neurology, The First Affiliated Hospital of Nanjing Medical UniversityNanjing, China; ^3^Department of Neurology, Meitan General HospitalXibahe Nanli, Beijing, China; ^4^Department of Physiology, Institute of Brain Research, School of Basic Medicine, Huazhong University of Science and TechnologyWuhan, China; ^5^Department of Biological Science, University at Albany, State University of New YorkAlbany, NY, USA; ^6^Department of Anesthesiology, Baylor College of MedicineHouston, TX, USA

**Keywords:** astrocytes, TREK-1 potassium channel, passive membrane conductance, patch-clamp recording, hippocampus

## Abstract

We have recently shown that a linear current-to-voltage (I-V) relationship of membrane conductance (passive conductance) reflects the intrinsic property of K^+^ channels in mature astrocytes. While passive conductance is known to underpin a highly negative and stable membrane potential (*V*_M_) essential for the basic homeostatic function of astrocytes, a complete repertoire of the involved K^+^ channels remains elusive. TREK-1 two-pore domain K^+^ channel (K_2P_) is highly expressed in astrocytes, and covalent association of TREK-1 with TWIK-1, another highly expressed astrocytic K_2P_, has been reported as a mechanism underlying the trafficking of heterodimer TWIK-1/TREK-1 channel to the membrane and contributing to astrocyte passive conductance. To decipher the individual contribution of TREK-1 and address whether the appearance of passive conductance is conditional to the co-expression of TWIK-1/TREK-1 in astrocytes, TREK-1 single and TWIK-1/TREK-1 double gene knockout mice were used in the present study. The relative quantity of mRNA encoding other astrocyte K^+^ channels, such as K_ir_4.1, K_ir_5.1, and TREK-2, was not altered in these gene knockout mice. Whole-cell recording from hippocampal astrocytes *in situ* revealed no detectable changes in astrocyte passive conductance, *V*_M_, or membrane input resistance (*R*_in_) in either kind of gene knockout mouse. Additionally, TREK-1 proteins were mainly located in the intracellular compartments of the hippocampus. Altogether, genetic deletion of TREK-1 alone or together with TWIK-1 produced no obvious alteration in the basic electrophysiological properties of hippocampal astrocytes. Thus, future research focusing on other K^+^ channels may shed light on this long-standing and important question in astrocyte physiology.

## Introduction

Mature hippocampal astrocytes exhibit a membrane K^+^ conductance characterized by a linear current-to-voltage (I-V) relationship (passive conductance), and a highly negative membrane potential (*V*_M_) (Zhou et al., 2006; Kafitz et al., 2008). Both of these electrophysiological features are fundamental for the basic homeostatic functions of astrocytes in the brain (Verkhratsky and Steinhauser, 2000; Walz, 2000; Olsen and Sontheimer, 2008; Wang and Bordey, 2008; Kimelberg, 2010).

While passive behavior and low membrane resistance (*R*_M_) represent intrinsic properties of membrane ion channels (Du et al., 2015), the molecular identity of K^+^ channels underlying this unique membrane conductance remains an issue to be fully resolved. Now, the inwardly rectifying K^+^ channel K_ir_4.1 and two-pore domain K^+^ channels (K_2P_) TWIK-1(K_2P_ 1.1) and TREK-1(K_2P_ 2.1) are considered the three major K^+^ channels, and the important contribution of K_ir_4.1 to astrocyte passive conductance has been well documented (Butt and Kalsi, 2006; Neusch et al., 2006; Djukic et al., 2007; Kucheryavykh et al., 2007; Seifert et al., 2009; Tong et al., 2014; Olsen et al., 2015).

In the hippocampus, K_ir_4.1 shows only moderate expression compared to other brain regions (Nwaobi et al., 2014). Consistently, our functional analysis indicated that 48% of passive conductance is mediated by K_ir_4.1 channels (Ma et al., 2014). To determine the contribution of other K^+^ channels to passive conductance, we initially considered TWIK-1 and TREK-1 as the two K_2P_ candidates based on an astrocyte transcriptome database (Cahoy et al., 2008; Zhou et al., 2009). However, TWIK-1 gene knockout affected the passive conductance minimally because of the retention of large amounts of TWIK-1 channels in intracellular compartments and the behavior of this channel as a non-selective cation channel in the membrane (Wang et al., 2013, 2015).

To explore a possible contribution of TREK-1 to passive conductance, TREK-1 gene knockout mice were introduced in this study. TREK-1 is an outwardly rectifying leak type K^+^ channel that follows Goldman-Hodgkin-Katz (GHK) constant field rectification (Fink et al., 1996). Thus, a combined expression of TREK-1 and K_ir_4.1 may hypothetically explain the observed passive conductance in astrocytes. TREK-1 is highly dynamic and subject to regulation by various changes occurring in the interstitial environment, such as pH, temperature, osmolarity, and mechanic force (Patel et al., 1998; Maingret et al., 1999; Lesage and Lazdunski, 2000; Chemin et al., 2005; Murbartián et al., 2005). This makes TREK-1 an attractive candidate as it may confer astrocytes’ ability to interact with neurons and integrate neuron-glia function.

The covalent association of TWIK-1/TREK-1 as a dimer via a disulfide bridge has been reported to underlie the trafficking of this heterodimer channel to the membrane and contribution to astrocyte passive conductance (Hwang et al., 2014). To address this question further, TWIK-1/TREK-1 double gene knockout mice were created to determine whether absence of both K_2P_ isoforms may affect the passive conductance.

We show that the basic electrophysiological properties of mature hippocampal astrocytes were not altered in either TREK-1 single or TWIK-1/TREK-1 double gene knockout mice, which calls attention to explore alternative K^+^ channels responsible for the still mysterious passive conductance in astrocytes.

## Materials and Methods

### Animals

All the experimental procedures were performed in accordance with a protocol approved by the Animal Care and Use Committees of The Ohio State University. TREK-1 gene (*Kcnk2*) knockout (TREK-1^−/−^) mice were generated on a mixed background of C57BL6J and SV129 strains. *Kcnk2* spans 136 Kbp of the mouse genome and has eight exons. As previously reported, for the mutant *Kcnk2*, a 4 Kbp genomic DNA sequence, from the position 14 bp of the second exon to the position 23 bp of the third intron, was replaced by a β-galactosidase/neomycin (LacZ/Neo) selection cassette (Namiranian et al., 2010). TWIK-1 gene (*Kcnk1*) knockout (TWIK-1^−/−^) mice were created with C57BL/6J genetic background, where the exon 2 of TWIK-1 gene (*Kcnk1*) was genetically deleted (Nie et al., 2005). TWIK-1/TREK-1 double knockout (TWIK-1^−/−^/TREK-1^−/−^) mice were generated by crossing TWIK-1^−/−^ and TREK-1^−/−^ mice.

Both male and female mice at postnatal day (P) of 21–28 were used, and the animals were divided into (1) wild type (WT); (2) TREK-1^−/−^; (3) TWIK-1^−/−^; and (4) TWIK-1^−/−^/TREK-1^−/−^ groups in this study.

### PCR Genotyping

To confirm the gene deletion, PCR genotyping was performed using total DNA isolated from tails of mice. Two pairs of primers were designed to recognize exon 3 of *Kcnk2* in WT mice and the LacZ/Neo selection cassette in TREK-1^−/−^ mice. TREK-1 WT primers: forward, GCTGGGTGAAGTTCTTCAGC; reverse, CATTACCTGGATGAGTTCGTC. TREK-1^−/−^ primers: forward, GCAGCGCATCGCCTTCTATC; reverse, AGGAGATGAAGACCTCTGCAAAGG. To detect the WT TWIK-1 gene (*Kcnk1*) and the truncated form of *Kcnk1* in TWIK-1^−/−^ mice, two pairs of primers were set to recognize exon 2 and to surround the exon 2, respectively. TWIK-1 WT primers: forward, TCCAGGGGAAGGCTACAA; reverse, GGAGACGGCAGGCAGTAA. TWIK-1^−/−^ primers: forward, CCCTTACTCTTATCAATCGG; reverse, GTTTGCTTGTGAATGTGC.

### RNA Extraction from Freshly Dissociated Single Astrocytes

Mice were anesthetized by intraperitoneal injection of 8% chloral hydrate in 0.9% NaCl saline (0.05 ml/100 g body weight). The brain was removed from the skull and placed in oxygenated (95% O_2_/5% CO_2_) Ca^2+^-free artificial cerebral spinal fluid (Ca^2+^-free aCSF; in mM):125 NaCl, 25 NaHCO_3_, 1.25 NaH_2_PO_4_, 3.5 KCl, 1 MgCl_2_, 1 Na-pyruvate, and 10 glucose (Zhou and Kimelberg, 2001; Xie et al., 2007). Coronal slices of 400 μm thickness were sectioned with a Vibratome (Pelco, 1500) and transferred to 34°C Ca^2+^-free aCSF supplemented with 1 μM SR101 (Invitrogen, New York, NY, USA; Nimmerjahn et al., 2004). After incubation with SR101 for 30 min, the brain slices were transferred to Ca^2+^-free aCSF for another 30 min at room temperature (20–22°C). Hippocampal regions were dissected out from the brain slices and digested for 25 min with 24 μ/ml papain in aCSF containing (in mM): 125 NaCl, 25 NaHCO_3_, 1.25 NaH_2_PO_4_, 3.5 KCl, 2 CaCl_2_, 1 MgCl_2_, and 10 glucose (osmolarity, 295 ± 5 mOsm; pH 7.3–7.4), supplemented with 0.8 mg/ml L-cysteine. After digestion, the hippocampal slices were returned to the Ca^2+^-free aCSF for recovery for at least 1 h. The loosened hippocampal slices were gently triturated into a cell suspension. To harvest single dissociated astrocytes, the cells were identified by their positive SR101 fluorescent staining and well-preserved soma and spatial domain (Bushong et al., 2002; Du et al., 2015). In each run, thirty astrocytes were collected for each genotype group by a glass electrode (diameter ~10 μm) attached to a micromanipulator. RNA extraction was done using RNeasy Mini Kit (Qiagen, Valencia, CA, USA) right after cell harvesting.

### Real-Time Quantitative Reverse Transcription PCR (QRT-PCR) Analysis

Immediately after RNA extraction, RNA was converted into cDNA using Applied Biosystem’s High Capacity cDNA Reverse Transcription Kit (Grand Islands, NY, USA). The PCR primer pairs for identification of TWIK-1, TREK-1, TREK-2, K_ir_4.1, K_ir_5.1, K_V_6.3 and GAPDH gene are shown in Table 1. All primer pairs spanned at least two exons to avoid amplification of genomic DNA. Each primer pair was tested by conventional PCR before quantitative reverse transcription PCR (qRT-PCR) to ensure that the amplicon yielded an anticipated and distinct single band. SYBR^®^ Select Master Mix (Invitrogen, New York, NY, USA) was used, and the qRT-PCR was run on a Step One Plus equipment (Life technologies, New York, NY, USA). Each assay was performed in triplicate samples from the same mouse. A minimum of three repeats were done for each genotype group. For each repetition, a negative control with no template was always present. *Gapdh* was used as the internal reference and routinely run in parallel with targeted genes. Data were obtained as Ct values (threshold cycle). The expression levels of target genes were expressed as 2^−ΔCT^, where ΔCT was referred to the Ct difference between gene of interest and *Gapdh*.

**Table 1:** **Primers for qRT-PCR analysis**.

Target gene		Primer sequence	Accession no
*Kcnk1* (TWIK-1)	F:	AATTGGAATTGGGACTTCAC	NM_008430.2
	R:	ACAGAGTAGATGATGCAGAAG	
*Kcnk2* (TREK-1)	F:	CCATCGGATTTGGAAACATCTC	NM_001159850.1
	R:	AAATGTGTCTTCCACTTTGG	
*Kcnk10* (TREK-2)	F:	AGTAGGCTTTGGTGATTTTG	NM_029911.4
	R:	CTCACCAACCTCTTCTTTTG	
*Kcnj10* (K_ir_4.1)	F:	GACAAACCCTTATCTGATTCC	NM_001039484.1
	R:	TGAGTCGTCTGACTGTAATAG	
*Kcnj16* (K_ir_5.1)	F:	GATCTGTAACTGAGGCTTTAAC	NM_001252207.1
	R:	AGGATAGCCTGGATATTTGG	
*Kcng4* (K_V_6.3)	F:	TTCTTCTCATTTCCCCCTAC	NM_025734.2
	R:	CTGTCGCTTGTTTTAGGAAG	
*Gapdh*	F:	AGGTTGTCTCCTGCGACTTCA	NM_008084.2
	R:	GTGGTCCAGGGTTTCTTACTCC	

*TWIK, a weakly inward rectifying K^+^ channel; TREK, a TWIK-related K^+^ channel; K_ir_, an inwardly rectifying K^+^ channel; K_V_, a voltage-gated K^+^ channel, Gapdh, glyceraldehyde-3-phosphatedehydrogenase. F, forward; R, reverse*.

### Preparation of Acute Hippocampal Slices

Hippocampal slices were prepared as we described previously (Ma et al., 2012a). Briefly, brains were rapidly removed from skulls and placed into ice-cold oxygenated (95% O_2_/5% CO_2_) slice cutting aCSF with reduced Ca^2+^ and increased Mg^2+^ (in mM): 125 NaCl, 3.5 KCl, 25 NaHCO_3_, 1.25 NaH_2_PO_4_, 0.1 CaCl_2_, 3 MgCl_2_ and 10 Glucose. Coronal hippocampal slices at 250 μm thickness were cut at 4°C and transferred to the oxygenated standard aCSF, recovering at room temperature for at least 1 h before SR101 incubation and electrophysiological recording.

### Electrophysiology

For *in situ* recording of astrocytes, individual hippocampal slices were transferred to the recording chamber mounted on an Olympus BX51WI microscope, with constant perfusion of oxygenated aCSF (osmolarity, 295 ± 5 mOsm; pH 7.3–7.4; 2.0 ml/min). Astrocytes located in the CA1 region were visualized using an infrared differential interference contrast (IR-DIC) video camera with the 40× water-immersion objective. The morphologically identified astrocytes could be further confirmed by their positive SR101 fluorescent staining. Whole-cell patch clamp recordings were performed using a MultiClamp 700A (or MultiClamp 700B) amplifier and pClamp 9.2 software (Molecular Devices, Sunnyvale, CA, USA). Borosilicate glass pipettes (Warner Instrument) were pulled from a Micropipette Puller (Model P-87, Sutter Instrument). The recording electrodes had a resistance of 3–7 MΩ when filled with the electrode solution containing (in mM): 140 K-gluconate, 13.4 Na-gluconate, 0.5 CaCl_2_, 1.0 MgCl_2_, 5 EGTA, 10 HEPES, 3 Mg-ATP, and 0.3 Na_2_-GTP (pH 7.23~7.25, 280 ± 5 mOsm). The electrode solution contained 3 mM low Cl^−^ and 14 mM high Na^+^, similar to physiological conditions (Kelly et al., 2009; Ma et al., 2012a).

The liquid junction potential was compensated for prior to forming the cell-attached mode in all recordings. After establishing whole-cell recording, the resting *V*_M_ was either read in “*I* = 0” mode or recorded under current clamp mode. In current clamp recording, the input resistance (*R*_in_) was measured by the built-in “Resistance test” function (63 pA/600 ms pulse) before and after recording. We have recently shown that mature hippocampal astrocytes exhibit a very low *R*_M_ of 6.4 MΩ, and a routinely achievable access resistance (*R*_a_) reading from “Membrane test” function is ~15 MΩ for astrocytes older than P21 (Zhou et al., 2009; Ma et al., 2014). Recordings with initial *R*_in_ greater than 20 MΩ or *R*_in_ varying more than 10% during recording were discarded.

In pharmacological experiments, 100 μM BaCl_2_ and 400 μM quinine were dissolved directly in aCSF for bath application to block astrocytic K_ir_4.1 and K_2P_s, respectively. The hypoosmotic bath solution, 205 ± 5 mOsm, was achieved by reducing 50 mM NaCl in normal aCSF. A temperature controller (Warner Instruments, TC-324C) was used to elevate the solution temperature in recording chamber to 32 ± 1°C. Unless noted specifically, most of the recordings were conducted at room temperature of ~22°C.

### Western Blot Analysis

#### Fractionation of Hippocampal Proteins from Different Subcellular Regions

Mice were anesthetized as described above, and then the hippocampus was quickly removed from anesthetized mice and separated into hydrophilic (cytoplasmic) and hydrophobic (membrane) proteins by Mem-PER Eukaryotic Membrane Protein Extraction Kit (Thermo Fisher Scientific, Rockford, IL, USA). To remove chemicals that may interfere with the following BCA protein assay and SDS-polyacrylamide gel electrophoresis (SDS-PAGE) analysis, all the fractioned protein samples were cleaned by SDS-PAGE Sample Prep Kit (Thermo Fisher Scientific, Rockford, IL, USA).

#### SDS-PAGE and Immunoblotting

Protein concentration was determined with the BCA protein assay kit (Thermo Fisher Scientific, Rockford, IL, USA). Samples were mixed with a 5× reducing loading buffer containing 100 mM DTT (Thermo Fisher Scientific, Rockford, IL, USA) and heated at 95°C for 5 min. Equal amounts of protein (25 μg/lane) were separated on a 4–12% tris-glycine gel (Bio-Rad, Hercules, CA, USA) and subsequently transferred to a nitrocellulose membrane (Micron Separations Inc., Westborough, MA). The membranes were blocked with 5% non-fat milk/TBST (Tris-buffered saline with 0.05% Tween 20) for 1 h at room temperature. The membranes were incubated with anti-TREK-1 antibodies (1:2000, Alomone Labs, Jerusalem, Israel) at 4°C overnight. After washing, the membranes were incubated with secondary antibody (Jackson ImmunoResearch Laboratories, Maine, USA). Immunoreactivity was detected with an enhanced chemiluminescent detection (Thermo Fisher Scientific, Rockford, IL, USA). Blots were scanned and quantified by Quantity One software (Bio-Rad, Hercules, CA, USA). After detecting TREK-1 immunoreactivity, the original membranes were stripped with stripping buffer (0.4 M Glycine, 0.2% SDS and 2% Tween 20, pH2.0) and re-probed with the following primary antibodies sequentially to determine the quality of protein fractionation: anti-Na^+^/K^+^ ATPase alpha 2(+) polypeptide (ATP1α2; 1:1000, Abgent, San Diego, CA, USA) and anti-glial fibrillary acidic protein (GFAP; 1:500, DAKO, Carpinteria, CA, USA). This antibody incubation and blot quantification followed the same procedure described above.

### Data Analysis

The patch clamp recording data were analyzed by Clampfit 9.2 (Molecular Devices, Sunnyvale, CA, USA) and Origin 8.0 (OriginLab, Northhampton, MA, USA). All the results are given as means ± SEM. Statistical analysis was performed using Student’s *t*-test of two independent samples. For multiple comparison tests, WT was used as control group; TWIK-1^−/−^, TREK-1^−/−^, and TWIK-1^−/−^/TREK-1^−/−^ were compared to against it. Thus, One-Way ANOVA followed by Dunnett’s test were combined for these tests. Significance level was set at *P* < 0.05.

## Results

### TREK-1 Single and TWIK-1/TREK-1 Double Gene Knockout Mice

**Figure 1:**
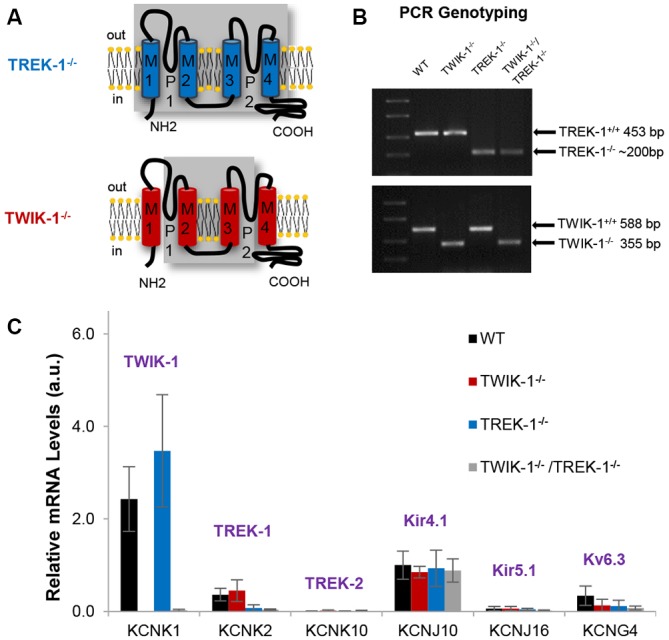
**Expression of astrocyte K^+^ channels in TREK-1, TWIK-1 single, and TWIK-1/TREK-1 double gene knockout mice. (A)** Schematic illustrations of genetic deletion of four transmembrane domains, including two pore-forming regions, in TREK-1 knockout (TREK-1^−/−^, upper), and TWIK-1 knockout (TWIK-1^−/−^, lower) mice. **(B)** PCR genotyping confirmation of successful genetic knockout of the targeted genes in TWIK-1^−/−^, TREK-1^−/−^, and TWIK-1/TREK-1 double knockout (TWIK-1^−/−^/TREK-1^−/−^) mice. **(C)** The relative quantity of mRNA of K^+^ channels in freshly dissociated mature astrocytes. The results were examined from wild type (WT), TWIK-1^−/−^, TREK-^1−/−^, and TWIK-1^−/−^/TREK-1^−/−^ astrocytes using qRT-PCR.

In TREK-1^−/−^ mice, four transmembrane domains, including two pore-forming regions, were genetically targeted for deletion as previously reported (Figure 1A, upper; Namiranian et al., 2010). The targeted deletion in TWIK-1^−/−^ mice included the second and third transmembrane domains (Figure 1A, lower; Nie et al., 2005; Wang et al., 2013). The targeted gene deletions were confirmed by PCR genotyping analysis. Specifically, to detect TREK-1^−/−^, one pair of primers was set to recognize exon 3 of WT *Kcnk2*, whereas another pair was set to recognize the LacZ-Neo selection cassette in the TREK-1 mutant. These resulted in the anticipated 453 bp for the WT *Kcnk2* and 200 bp for the *Kcnk2* mutant, respectively (Figure 1B, upper; Namiranian et al., 2010). To detect the truncated *Kcnk1* in TWIK-1^−/−^, one pair of primers was set to recognize exon 2 in WT, and another pair was set to surround exon 2. These resulted in the anticipated bands of 588 bp and 355 bp in amplicons (Figure 1B, lower; Wang et al., 2013). TWIK-1^−/−^/TREK-1^−/−^ showed an anticipated band at ~200 bp for *Kcnk2* mutant as well as a 355 bp band for the truncated *Kcnk1* (Figure 1B).

As reported previously, the growth, fertility and gross anatomy of TREK-1^−/−^ and TWIK-1^−/−^ do not differ from their age- and gender-matched WT mice, and their offspring from heterozygote mating followed a Mendelian ratio (Nie et al., 2005; Namiranian et al., 2010; Wang et al., 2013). TWIK-1^−/−^/TREK-1^−/−^ mice were generated by crossing two mouse lines. The resulted mice essentially exhibit similar characteristics as the WT mice, and their offspring from heterozygote mating followed a Mendelian ratio.

### TREK-1 and TWIK-1 Gene Knockout does not Alter the Expression of Other Astrocyte K^+^ Channels at Trascript Levels

To clarify whether single TREK-1, TWIK-1, or double TWIK-1/TREK-1 gene knockout would interfere with the expression of the other astrocyte K^+^ channels or produce compensation for the loss of these two K^+^ channels, we compared the expression levels of mRNA for a group of K^+^ channels that were selected based on the following considerations. First, we included the three major astrocyte K^+^ channels known to express relative mRNA levels of TWIK-1 > K_ir_4.1 > TREK-1 in isolated cortical astrocytes (Cahoy et al., 2008) and freshly isolated hippocampal astrocytes (Seifert et al., 2009). Second, for other K_2P_ members, we selected TREK-2 in the TREK subfamily, as it distributes extensively in the central nervous system (CNS) and its mRNA expression overlaps with TREK-1 expression in many brain regions (Medhurst et al., 2001; Talley et al., 2001). As TWIK-2 and TWIK-3 in the TWIK subfamily were undetectable in our previously study in both WT and TWIK-1^−/−^ mice (Wang et al., 2013), these two K_2P_ isoforms were not included in this analysis. Third, we included K_ir_5.1 and K_V_6.3, which showed relatively high astrocytic expression among other K^+^ channels in K_ir_ and K_V_ subfamilies, respectively (Hibino et al., 2004; Cahoy et al., 2008).

In isolated WT hippocampal astrocytes, qRT-PCR analysis revealed an order of mRNA expression levels of TWIK-1 > K_ir_4.1 > TREK-1 > K_V_6.3. TREK-2 and K_ir_5.1 were barely detectable. In knockout mice, qRT-PCR results confirmed the anticipated deletion of TREK-1 and TWIK-1 either alone or together (Figure 1C). Furthermore, in all tested genotypes, the expression levels and the relative abundance of other astrocyte K^+^ channels were not altered compared to WT. Thus, targeted TREK-1 and TWIK-1 gene deletion, either alone or together, did not result in an altered profile of K^+^ channel expression, nor compensatory up-regulation in any of the candidate K^+^ channels (Figure 1C). Nevertheless, these results could not fully rule out a potential compensation of passive conductance by other unknown K^+^ channels in TREK-1 and/or TWIK-1 gene knockout mice.

In summary, the anticipated single and double TWIK-1 and TREK-1 gene knockouts were successful in the mouse lines used in this study, and these gene deletions did not alter the expression pattern of other known astrocytic K^+^ channels that have the potential to functionally compensate for changes in astrocyte passive conductance.

### TREK-1 Gene Knockout does not Alter the Basic Electrophysiological Properties of Mature Hippocampal Astrocytes

TREK-1 follows the GHK prediction for a leak type K^+^ channel by showing an outwardly rectifying current profile in an asymmetrical physiological K^+^ gradient (Fink et al., 1996; Enyedi and Czirják, 2010). As a leak type K^+^ channel, the contribution of TREK-1 to the resting *V*_M_ was unequivocally demonstrated in a human adrenal cell line (Brenner and O’Shaughnessy, 2008).

**Figure 2:**
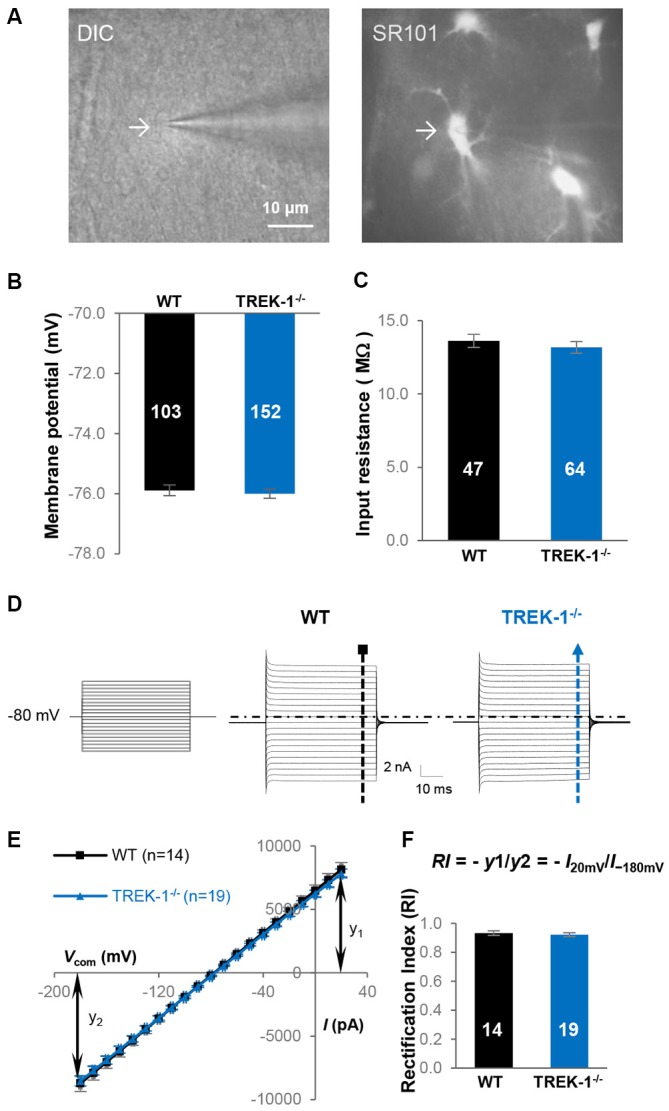
**TREK-1 gene deletion does not alter the electrophysiological properties of astrocytes. (A)** Identification of astrocyte in hippocampal slices based on cellular morphology and positive SR101 staining in CA1 region. **(B,C)** Membrane potential (*V*_M_), and input resistance (*R*_in_) in WT and TREK-1^−/−^ astrocytes. **(D)** Representative astrocyte whole-cell passive conductance from a WT and a TREK-1^−/−^ astrocyte are shown separately as indicated. The voltage commands used for whole-cell current induction are displayed on the left panel. **(E)** I-V plots show the averaged current amplitudes from WT and TREK-1^−/−^ astrocytes. The equation for rectification index (RI) is also illustrated and the resulted RI values are summarized in **(F)**.

To determine the contribution of TREK-1 to astrocyte membrane conductance and resting *V*_M_, whole-cell patch clamp recordings were made from mature hippocampal CA1 astrocytes from WT and TREK-1^−/−^ mice (Figure 2A). The whole-cell resting *V*_M_ was comparable between the two genotypes: −75.9 ± 0.17 mV (*n* = 103) in WT *vs*. −76.0 ± 0.15 mV (*n* = 152) in TREK-1^−/−^ (*P* > 0.05, Figure 2B), so was the *R*_in_: 13.6 ± 0.45 MΩ (*n* = 47) in WT *vs*. 13.2 ± 0.40 MΩ (*n* = 64) in TREK-1^−/−^ (*P* > 0.05, Figure 2C). We have previously shown that passive membrane conductance exhibits a weak inward rectification with a rectification index (RI) around 0.9 (Wang et al., 2013). Should the outwardly rectifying TREK-1 contribute substantially to passive conductance, TREK-1^−/−^ would increase the inward rectification of whole-cell current with a further reduced RI value. However, the RI was essentially unchanged in TREK-1^−/−^ astrocytes: 0.93 ± 0.02 (*n* = 14) in WT *vs*. 0.93 ± 0.01 (*n* = 19) in TREK-1^−/−^ (*P* > 0.05, Figures 2D–F). Altogether, basic electrophysiological features remain intact in TREK-1^−/−^ astrocytes.

In hippocampal astrocytes, K_ir_4.1 contributes to 48% of passive membrane conductance (Ma et al., 2014), therefore inhibition of K_ir_4.1 should improve voltage clamping quality for a higher resolution examination of TREK-1 conductance using RI analysis. Accordingly, K_ir_4.1 was inhibited by 100 μM BaCl_2_ (IC_50_ ~ 3.5 μM; Ransom and Sontheimer, 1995; Seifert et al., 2009), and RI was compared again between WT and TREK-1^−/−^ astrocytes.

**Figure 3:**
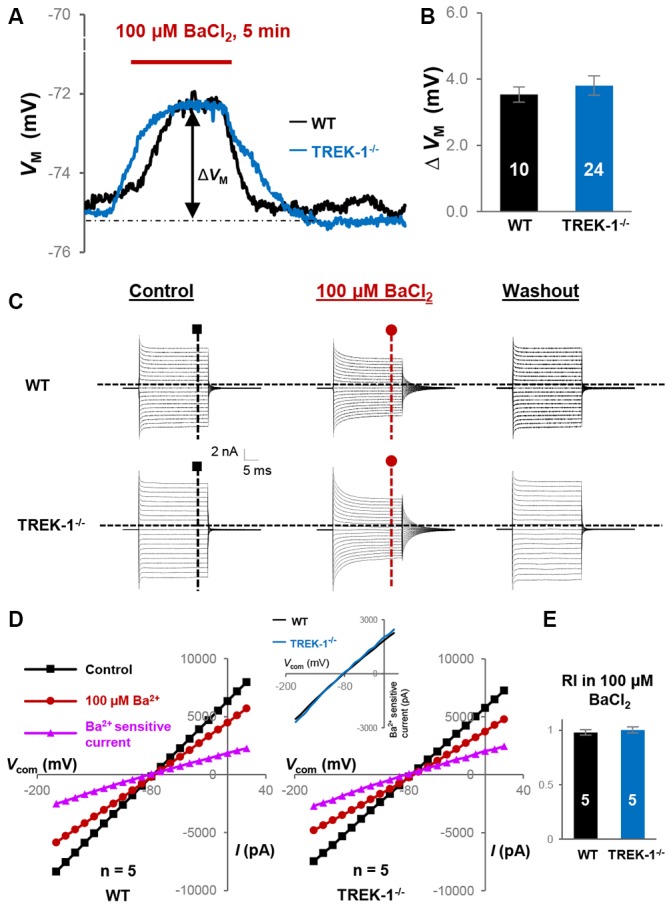
**K_ir4.1_ inhibition does not reveal the functional contribution of TREK-1 in TREK-1^−/−^ astrocytes. (A)** Representative *V*_M_ response to K_ir_4.1 inhibitor 100 μM BaCl_2_ from a WT and a TREK-1^−/−^ astrocyte, as indicated *in situ*. Δ*V*_M_ indicates the peak *V*_M_ depolarization during a 5 min BaCl_2_ bath application. **(B)** Summary of 100 μM BaCl_2_-induced *V*_M_ depolarization, where the *V*_M_ depolarization was comparable between WT and TREK-1^−/−^ astrocytes. **(C)** Representative whole-cell current recorded first in control, then 5 min in 100 μM BaCl_2_, and washout. **(D)** I-V plots derived from recordings in **(C)**. The Ba^2+^-sensitive currents in I-V plots were obtained from sweep subtraction. The Ba^2+^-sensitive currents were shown in expanded *y*-axis in the inset that showed a moderate inward rectification in both WT, RI = 0.91, and TREK-1 KO, RI = 0.90, respectively. **(E)** Summary of RI values from WT and TREK-1^−/−^ astrocytes obtained from recordings in the presence of 100 μM BaCl_2_ for K_ir_4.1 inhibition; the RI values were comparable between the two groups.

In current clamp recording, 100 μM BaCl_2_-induced *V*_M_ depolarization did not differ significantly between WT, 3.5 ± 0.23 mV (*n* = 10), and TREK-1^−/−^ astrocytes, 3.8 ± 0.29 mV (*n* = 24; *P* > 0.05, Figure 3B). This result was consistent with an unchanged K_ir_4.1 expression at mRNA levels in TREK-1^−/−^ mice (Figure 1C). In voltage clamp recording, 100 μM BaCl_2_ suppressed the whole-cell passive conductance comparably in WT and TREK-1^−/−^ astrocytes, and the resulting Ba^2+^-sensitive currents were also comparable (Figures 3C,D). However, inhibition of K_ir_4.1 shifted RI toward a linear level, i.e., approach to the value 1, in both WT and TREK-1^−/−^ astrocytes: 0.98 ± 0.03 (*n* = 5) in WT *vs*. 1.00 ± 0.03 (*n* = 5) in TREK-1^−/−^ astrocytes (*P* > 0.05, Figure 3E), which further corroborates the view that TREK-1 shows no detectable contribution to passive conductance.

Interestingly, Ba^2+^-induced linear shift in RI differed significantly when compared with control groups both in WT and TREK-1^−/−^ astrocytes (*P* < 0.05, data not shown), indicating elimination of a significant portion of K_ir_4.1-mediated inward currents from the overall passive conductance.

The activity of TREK-1 channel can be positively modulated by hypoosmotic extracellular solution and warmer temperature in the heterologous expression system (Maingret et al., 1999, 2000). To explore whether the same conditions could facilitate identification of functional TREK-1 in hippocampal astrocytes, we first applied hypoosmotic aCSF, −90 mOsm, to astrocyte recordings. However, the RI remained comparable between WT (0.95 ± 0.01, *n* = 3) and TREK-1^−/−^ astrocytes (0.95 ± 0.02, *n* = 4; *P* > 0.05). Raising the temperature of the bath aCSF to 32 ± 1°C did not alter the RI: 0.95 ± 0.01 (*n* = 6) in WT *vs*. 0.95 ± 0.02 (*n* = 3) in TREK-1^−/−^ astrocytes (*P* > 0.05). Both in WT and in TREK-1^−/−^ astrocytes, the RI values in high temperature were comparable to the RI in room temperature. Additionally, arachidonic acid, halothane and angiotensin II have been shown as TREK-1 modulators (Fink et al., 1998; Patel et al., 1999; Enyeart et al., 2005). However, none of these TREK-1 modulators induced noticeable change in astrocyte RI, *V*_M_, and the amplitude of passive conductance in WT astrocytes (data not shown).

Together, these results show that functional K_ir_4.1 expression is largely intact in TREK-1^−/−^ astrocytes, and there is no detectable contribution of TREK-1 to astrocyte passive conductance.

### TREK-1 Channels are Predominantly Located in Cytoplasm in Hippocampus

**Figure 4:**
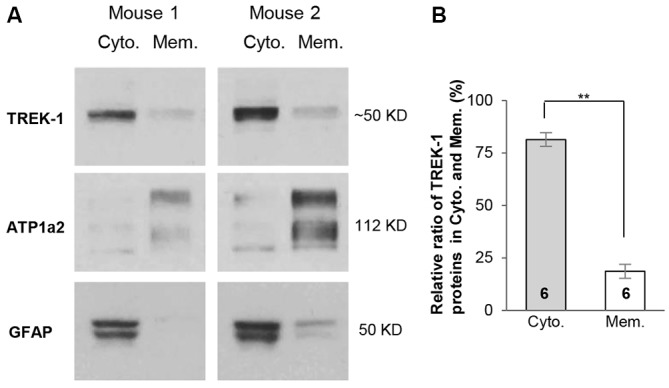
**TREK-1 channels are predominantly located in cytoplasm. (A)** Fractionation western blot results revealed the subcellular distribution of TREK-1 channels in cytoplasmic fraction *vs*. membrane fraction in two independent tests of mice hippocampal samples. Anti-glial fibrillary acidic protein (GFAP) (50 kDa) and ATP1α2 (112 kDa) were markers for cytoplasmic and membrane fractions, respectively. The blots shown in **(A)** were first incubated with anti-TREK-1 antibody and then re-probed with the rest of other primary antibodies sequentially after the original membranes were stripped with stripping buffer (see “Materials and Methods” Section). **(B)** Bar graph summary showing the relative ratio of TREK-1 proteins located in cytoplasmic *vs*. membrane fractions. Data are shown as mean ± SEM. Numbers indicate the times of observations. **p < 0.01.

Because TREK-1 gene knockout does not affect the basic properties of astrocytes, we explored further the mechanism underlying this observation. We have previously shown that TWIK-1 proteins are mainly located in the intracellular compartments of hippocampal astrocytes (Wang et al., 2013), whereas the subcellular distribution of TREK-1 in hippocampal astrocytes is yet unknown. Differing from TWIK-1, TREK-1 is widely expressed in neurons and astrocytes in the CNS, and the subcellular distribution of this channel varies substantially depending on different species, tissues and cell types (Hervieu et al., 2001; Medhurst et al., 2001; Talley et al., 2001). To address whether lack of functional TREK-1 contribution in astrocytes is also attributable to retention of channels in the intracellular compartments, cytoplasmic and membrane proteins were extracted separately from hippocampus in fractionation western blot analysis as we reported previously (Wang et al., 2013). In this analysis, the antibodies against ATP1α2 and GFAP were used as astrocyte-specific membrane and cytoplasmic markers (Eng et al., 2000; Dinuzzo et al., 2012; Wang et al., 2013). Similar to TWIK-1 proteins, TREK-1 proteins showed a predominant location in the cytoplasmic fraction (Figure 4A). Specifically, 81.4 ± 3.3% of the total TREK-1 proteins were located in the cytoplasmic fraction in contrast with 18.6 ± 3.3% of channel proteins in the membrane fraction (*n* = 6, *P* < 0.01, Figure 4B). As TREK-1 is expressed both in astrocytes and neurons, it remained unknown whether TREK-1 expression in the membrane fraction is astrocytic, neuronal, or shared by both. Nevertheless, the results suggest that retention of TREK-1 in the intracellular compartments is likely accountable for the limited functional contribution of TREK-1 to astrocyte membrane conductance and *V*_M_.

### TWIK-1/TREK-1 Double Gene Knockout does not Alter Astrocyte Passive Conductance

We have previously shown that membrane TWIK-1 functions as a non-selective cation channel with no detectable contribution to passive conductance (Wang et al., 2013). Here, we show no detectable functional contribution of TREK-1 to passive conductance. However, formation of a TWIK-1/TREK-1 heterodimer via a disulfide bridge has been shown as a prerequisite for trafficking of the TWIK-1/TREK-1 heterodimer to the membrane and contribution to astrocyte passive conductance (Hwang et al., 2014). In the very same study, *in vivo* delivery of shRNA for knockdown of either TWIK-1 or TREK-1 was sufficient enough to eliminate passive conductance (Hwang et al., 2014). To answer whether co-expression of TWIK-1 and TREK-1 is required for astrocytes to show passive conductance, we examined the impact of TWIK-1^−/−^/TREK-1^−/−^ on the basic electrophysiological properties of astrocytes.

**Figure 5:**
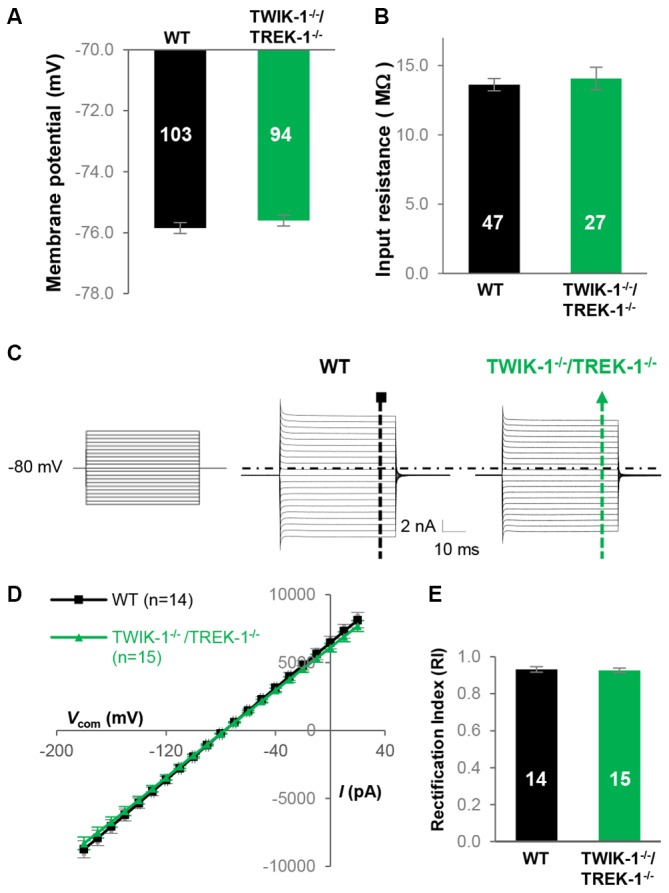
**Genetic deletion of TWIK-1 and TREK-1 genes together does not alter the electrophysiological properties of astrocytes. (A,B)** Bar graph summary of the *V*_M_ and *R*_in_ from WT and TWIK-1^−/−^/TREK-1^−/−^ astrocytes. **(C)** Representative whole-cell current profiles from WT and TWIK-1^−/−^/TREK-1^−/−^ astrocytes, respectively. **(D)** Averaged I-V plots from these two genotypes, where the whole-cell current amplitudes in both inward and outward directions were comparable. **(E)** The RI values were also comparable between the two genotypes.

We show that the *V*_M_ was comparable between the two experimental groups: −75.9 ± 0.17 mV (*n* = 103) in WT *vs*. −75.7 ± 0.18 mV (*n* = 94) in TWIK-1^−/−^/TREK-1^−/−^ astrocytes (*P* > 0.05; Figure 5A), indicating a TWIK-1/TREK-1 heterodimer is not required for the highly negative *V*_M_ that is critical for the homeostatic function of astrocytes (Figure 5A). Second, the *R*_in_ was similar between WT, 13.6 ± 0.45 MΩ (*n* = 47), and TWIK-1^−/−^/TREK-1^−/−^, 14.1 ± 0.80 MΩ (*n* = 27) (*P* > 0.05; Figure 5B), indicating no change in the amount of background leak channels in the absence of two highly expressed leak type K^+^ channels, TWIK-1 and TREK-1, in astrocytes. Third, in the absence of TWIK-1 and TREK-1, the former a weakly inward rectifier and the latter a GHK outwardly rectifier channel (Fink et al., 1996; Lesage et al., 1996a), the RI was essentially unchanged in astrocytes: 0.94 ± 0.01 (*n* = 15) in TWIK-1^−/−^/TREK-1^−/−^ compared to 0.93 ± 0.02 (*n* = 14) in WT (*P* > 0.05, Figures 5C–E).

**Figure 6:**
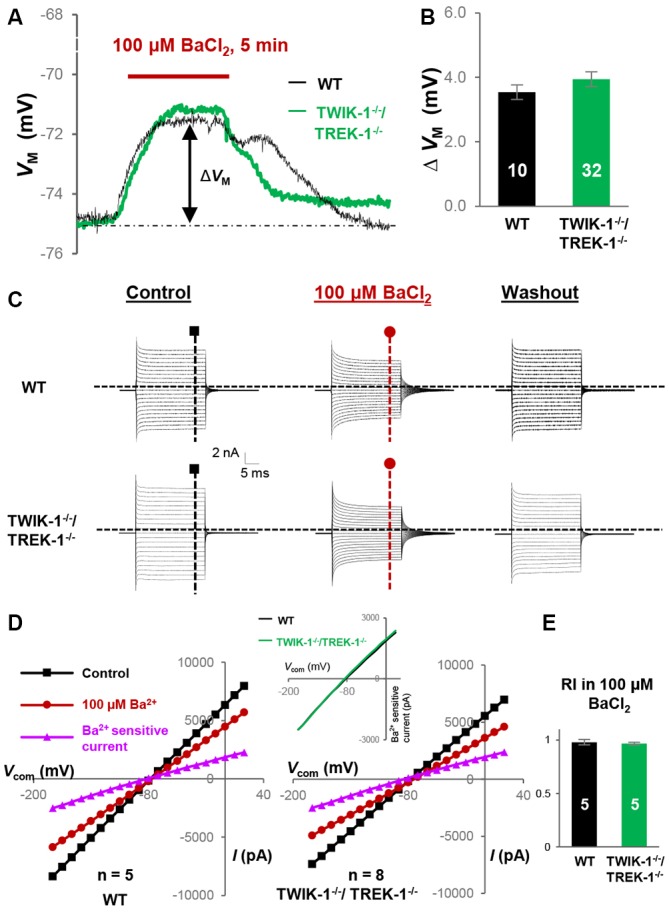
**K_ir_4.1 inhibition does not reveal the functional contribution of TWIK-1/TREK-1 in double gene knockout mice. (A)** Representative *V*_M_ response to K_ir_4.1 inhibitor, 100 μM BaCl_2_, from a WT and a TWIK-1^−/−^/TREK-1^−/−^ astrocyte as indicated *in situ*. **(B)** Summary of 100 μM BaCl_2_-induced *V*_M_ depolarization, where the *V*_M_ depolarization was comparable between WT and TWIK-1^−/−^/TREK-1^−/−^ astrocytes. **(C)** Representative whole-cell current recorded first in control, then 5 min in 100 μM BaCl_2_, and washout. I–V relationships were shown in **(D)**. **(D)** I–V plots derived from recordings in **(C)**. The Ba^2+^-sensitive currents, in I-V plots were obtained from sweep subtraction. The Ba^2+^- sensitive currents were shown in expanded *y*-axis in the inset that showed a moderate inward rectification in both WT, RI = 0.91, and double gene knockout mice, RI = 0.90, respectively. **(E)** Summary of RI values from WT and TWIK-1^−/−^/TREK-1^−/−^ astrocytes obtained from recordings in the presence of 100 μM BaCl_2_ for K_ir_4.1 inhibition; the RI values were comparable between the two groups.

Consistent with our mRNA analysis, where K_ir_4.1 expression was unchanged in TWIK-1^−/−^/TREK-1^−/−^ astrocytes (Figure 1C), 100 μM BaCl_2_-induced *V*_M_ depolarization was comparable between WT, 3.5 ± 0.23 mV (*n* = 10), and TWIK-1^−/−^/TREK-1^−/−^ astrocytes, 3.9 ± 0.23 mV (*n* = 32; *P* > 0.05, Figures 6A,B). In voltage clamp recording, 100 μM BaCl_2_ produced similar inhibition of passive conductance as compared with WT and TWIK-1^−/−^/TREK-1^−/−^ astrocytes (Figures 6C,D). Additionally, in the presence of 100 μM BaCl_2_, the RI of Ba^2+^-insensitive currents was comparable between WT and TWIK-1^−/−^/TREK-1^−/−^ astrocytes: 0.98 ± 0.03 (*n* = 5) in WT *vs*. 0.97 ± 0.01 (*n* = 5) in TWIK-1^−/−^/TREK-1^−/−^ astrocytes (*P* > 0.05, Figure 6E). These results indicated that neither TWIK-1 nor TREK-1 has a significant contribution to the remaining whole-cell currents in the absence of K_ir_4.1 channels.

**Figure 7:**
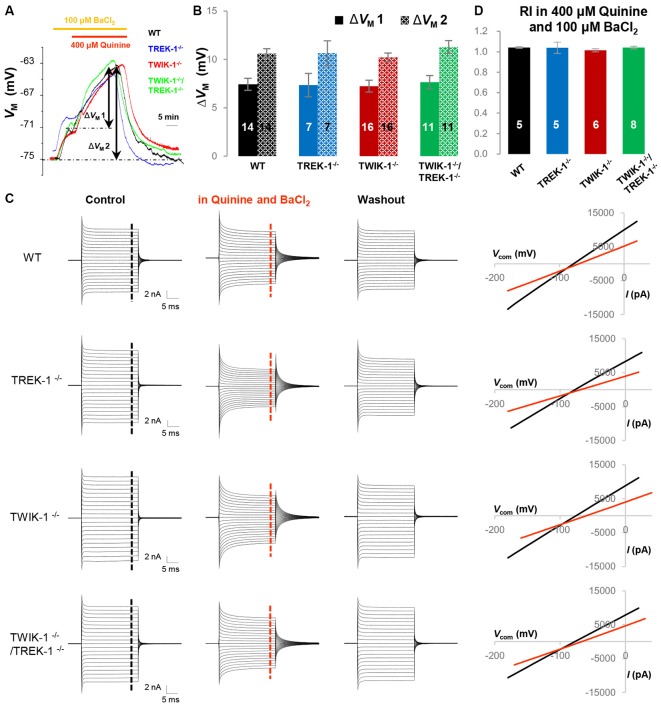
**Quinine does not reveal the functional contribution of TWIK-1 and TREK-1 in single or double gene knockout mice. (A)** Representative astrocyte *V*_M_ recordings first in 100 μM BaCl_2_ bath application for 5 min, followed by addition of 400 μM quinine for 20 min, from a WT, TREK-1^−/−^, TWIK-1^−/−^ and TWIK-1^−/−^/TREK-1^−/−^ astrocyte as indicated *in situ*. **(B)** Summary of 400 μM quinine-induced *V*_M_ depolarization (Δ*V*_M_ 1) and the total *V*_M_ depolarization induced by BaCl_2_ plus quinine from all four genotypes. **(C)** Representative whole-cell current recordings first in aCSF as control, then in 100 μM BaCl_2_ plus 400 μM quinine, and washout. Representative I–V relationships were shown in the right panel. **(D)** Summary of RI values from four genotypes obtained from astrocyte recordings in the presence of 400 μM quinine and 100 μM BaCl_2_ together in bath. The RI values were comparable among the four genotypes.

Quinine is a potent TWIK-1 and TREK-1 inhibitor (Zhou et al., 2009). To examine and compare K_2P_ conductance selectively in gene knockout astrocytes, the K_ir_4.1 was first inhibited by 100 μM BaCl_2_, and that was followed by addition of 400 μM quinine. Under these conditions, quinine further depolarized astrocyte *V*_M_ (Δ*V*_M_1) by 7.4 ± 0.50 mV (*n* = 14) in WT, 7.4 ± 1.30 mV (*n* = 7) in TREK-1^−/−^, 7.2 ± 0.44 mV (*n* = 16) in TWIK-1^−/−^ and 7.4 ± 0.68 mV (*n* = 11) in TWIK-1^−/−^/TREK-1^−/−^ astrocytes (*P* > 0.05, Figures 7A,B). Total *V*_M_ depolarization (Δ*V*_M_2) induced by 100 μM BaCl_2_ and 400 μM quinine was also comparable in the four genotypes: 10.6 ± 0.62 mV (*n* = 14) in WT, 10.6 ± 1. 20 mV (*n* = 7) in TREK-1^−/−^, 10.6 ± 0.62 mV (*n* = 16) in TWIK-1^−/−^ and 11.0 ± 0.69 mV (*n* = 11) in TWIK-1^−/−^/TREK-1^−/−^ astrocytes (*P* > 0.05, Figures 7A,B). In voltage clamp recording, 400 μM quinine produced similar inhibition of passive conductance as compared with WT, TREK-1^−/−^, TWIK-1^−/−^ and TWIK-1^−/−^/TREK-1^−/−^ astrocytes (Figure 7C). Specifically, in the presence of 400 μM quinine and 100 μM BaCl_2_, the RI of quinine and Ba^2+^-insensitive currents was comparable between WT, TREK-1^−/−^, TWIK-1^−/−^ and TWIK-1^−/−^/TREK-1^−/−^ astrocytes: 1.04 ± 0.01 (*n* = 5) in WT, 1.04 ± 0.06 (*n* = 5) in TREK-1^−/−^, 1.03 ± 0.02 (*n* = 6) in TWIK-1^−/−^ and 1.04 ± 0.01 (*n* = 8) in TWIK-1^−/−^/TREK-1^−/−^ astrocytes (*P* > 0.05, Figure 7D). These results strongly indicate that quinine sensitive K_2P_ conductance is not altered in gene knockout astrocytes.

In summary, by using genetic gene knockout approach, our study found no evidence in support of the notion that the TWIK-1/TREK-1 heterodimer contributes to the passive conductance of mature hippocampal astrocytes.

## Discussion

The present study was conceived to determine the contribution of TREK-1 to astrocyte passive conductance and resting *V*_M_, and to answer whether co-expression of TWIK-1/TREK-1, presumably as heterodimer channel, is required for their functional contribution to passive conductance. To address these questions, TREK-1 single- and TWIK-1/TREK-1 double-gene knockout mice were used in this study. We found that the passive conductance and the resting *V*_M_ of mature hippocampal astrocytes were not noticeably altered in these gene knockout mice. Additionally, similar to TWIK-1(Wang et al., 2013), TREK-1 proteins were retained predominantly in the intracellular compartments, which likely accounts for the limited TREK-1 and TWIK-1 contribution, either alone or together, to the basic electrophysiological properties of mature hippocampal astrocytes.

### Passive Conductance and Astrocyte Function

Increasing evidence shows that astrocytes act as the most versatile cell type in various CNS functions such as CNS homeostasis, synaptogenesis, synaptic transmission modulation, and neurovascular signaling (Nedergaard et al., 2003; Haydon and Carmignoto, 2006; Barres, 2008; Wang and Bordey, 2008; Zolessi, 2009; Kimelberg, 2010). Among them, the homeostatic support function was the earliest role assigned to astrocytes. The discovery of ohmic behavior passive conductance in amphibian optic nerve neuroglia was one of the cornerstones that led to this notion (Kuffler et al., 1966) and also promoted the “K^+^ spatial buffering hypothesis” (Orkand et al., 1966). Afterward, the importance of this conductance in astrocyte homeostatic functions, such as neurotransmitter uptake and pH regulation, has been increasingly appreciated (Wang and Bordey, 2008; Kimelberg, 2010).

At the mRNA expression level, TWIK-1, K_ir_4.1 and TREK-1 are the highly expressed K^+^ channels in isolated cortical astrocytes (Cahoy et al., 2008). While the inwardly rectifying K_ir_4.1 has been a well-recognized and studied astrocytic K^+^ channel (Olsen et al., 2015) at both protein and functional levels, K_ir_4.1 expression is relatively low in mouse hippocampal astrocytes compared to its expression in the spinal cord and brainstem (Ma et al., 2014; Nwaobi et al., 2014). This leaves the molecular identities of other K^+^ channels in hippocampal astrocytes to be further determined.

In the heterologous system, constitutive endocytosis restrains TWIK-1 to the recycling endosomes, and membrane TWIK-1 is able to conduct Na^+^ in the event of a fall in extracellular pH or K^+^ concentration (Feliciangeli et al., 2010; Chatelain et al., 2012; Ma et al., 2012c). In native cells, i.e., astrocytes, pancreatic β cells, and kidney tubular cells, TWIK-1 behaves as a non-selective cation channel (Millar et al., 2006; Chatelain et al., 2012; Wang et al., 2013). Because the channel is also highly permeable to ${\text{NH}}_{4}^{+}$ (Ma et al., 2012b), a critical role of astrocytic TWIK-1 in ${\text{NH}}_{4}^{+}$ homeostasis has been recently identified (Wang et al., 2015). These findings make it less likely that TWIK-1 could act as a classic background K^+^ channel in astrocytes. Thus, here we extended the search to another K_2P_ channel, TREK-1.

### Absence of Fucntional Trek-1 Contribution to Astrocyte Passive Conductance and Resting *V*_M_

TREK-1 is highly expressed in astrocytes and neurons in the mouse CNS (Meadows et al., 2000; Nicolas et al., 2004). Although TREK-1 shares a similar overall structural arrangement with TWIK-1, consisting of two pore-forming domains and four transmembrane segments (TMS), this structural similarity does not give rise to similar electrophysiological characteristics. Specifically, TWIK-1 in heterologous systems is characterized by a weakly inward rectification, whereas TREK-1 is an outward rectifier following GHK constant field rectification in physiological K^+^ solutions (Fink et al., 1996; Lesage et al., 1996a). TREK-1 is responsive to a variety of physiochemical stimuli and pharmacological agents, such as changes in temperature, osmolarity, mechanical force and anesthetic. The channel activity can also be modulated by activation of Gs and Gq coupled receptors (Patel et al., 1998; Maingret et al., 1999; Lesage and Lazdunski, 2000; Chemin et al., 2005; Murbartián et al., 2005). Thus, TREK-1 has the potential to enable astrocytes to engage in astrocyte-neuron crosstalk through a unique channel mechanism.

In the present study, TREK-1 gene knockout resulted in no detectable change in the basic electrophysiological properties in hippocampal astrocytes. First, the macroscopic whole-cell passive conductance and resting *V*_M_ were not altered in mature astrocytes of TREK-1^−/−^ mice. Second, the *R*_in_, a functional readout of the quantity of resting open leak channels, did not differ between WT and TREK-1^−/−^ astrocytes. Third, the RI remained comparable between WT and TREK-1^−/−^ astrocytes when K_ir_4.1 was pharmacologically inhibited by 100 μM Ba^2+^. Fourth, when K_ir_4.1 was inhibited by 100 μM Ba^2+^, the putative quinine-sensitive K_2P_ conductance and quinine induced *V*_M_ depolarization remained comparable between WT and TREK-1^−/−^ astrocytes.

Interestingly, qRT-PCR analysis from other K^+^ channels showing variable expression levels in WT astrocytes (Cahoy et al., 2008) resulted in no detectable change in those candidate channels in TREK-1^−/−^ astrocytes; thus compensation by other K^+^ channels for the loss of TREK-1 is unlikely to occur in knockout astrocytes.

### Subcellular Location of Trek-1 Protein in Hippocampus

The subcellular distribution of TREK-1 appears to be quite diverse. In the rat brain TREK-1 is predominantly expressed in the plasma membrane of projection neurons and interneurons (Hervieu et al., 2001), whereas the same channel in human HaCaT cell line is widespread in various subcellular locations, including the perinuclear, nuclear compartments and other intracellular organelles (Kang et al., 2007).

Interestingly, TREK-1e, a nonfunctional splice variant of the TREK-1 channel resulting from the skipping of exon 5 in gene transcription and subsequent loss of the second pore-forming domain between the transmembrane domains M3 and M4, was identified from human and rat brain and kidney (Rinne et al., 2014). In transfected COS-7 cells, the TREK-1e expression appears to be retained in the endoplasmic reticulum (ER) with a potential role of modulating vesicular traffic of full-length TREK-1 channels from the ER to the plasma membrane (Rinne et al., 2014).

In the present study, a preferential cytoplasmic expression was observed for TREK-1 channels expressed in the hippocampus (Figures 3A,B). The antibody used in our western blot analysis was generated to recognize the N-terminus of TREK-1, which includes the splice variant TREK-1e. Although more precise analysis with the TREK-1e specific antibody is needed to explore this issue further, should TREK-1e be expressed in astrocytes, one explanation for lack of functional TREK-1 contribution to passive conductance would be the retention of TREK-1 in the ER.

As noted, 18.6% of TREK-1 protein in the membrane fraction could be neuronal, astrocytic or both. Therefore, we are yet unable to determine the precise membrane presence of TREK-1 in astrocytes. Nevertheless, passive conductance and astrocyte *V*_M_ was unchanged in the presence of several TREK-1 activators, such as arachidonic acid, halothane (data not shown); thus it is possible that the membrane expression of TREK-1 proteins may be predominantly neuronal in mice hippocampus, which offers another plausible explanation for lack of functional TREK-1 contribution.

TREK-1 trafficking and targeting to the plasma membrane is subject to regulation through several identified mechanisms. First, TREK-1e might modulate vesicular traffic of full-length TREK-1 channels from the ER to the plasma membrane in transfected COS-7 cells (Rinne et al., 2014). Second, TREK-1 has a distinct C terminus binding site with microtubule associated protein (Mtap2) in neurons, resulting in an enhanced membrane TREK-1 expression and functional currents (Sandoz et al., 2008). Third, β_IV_-spectrin, an actin-associated protein, is required for the membrane targeting and activity of TREK-1 in the cardiomyocytes (Hund et al., 2014). It remains to be determined whether any of these mechanisms exist in astrocytes.

### Astrocyte Passive Conductance Remains Intact in the Absence of TWIK-1/TREK-1 K^+^ Channels

Most of the functional K_2P_ channels are formed by covalent association of two K_2P_ subunits, and each subunit contributes to two pore-forming regions of K^+^ selective filter (Lesage et al., 1996b; Doyle et al., 1998). It has been shown that formation of a TWIK-1/TREK-1 heterodimeric channel is the mechanism underlying astrocytic passive conductance (Hwang et al., 2014). In the present study, we have addressed this possibility through a different approach; by genetic elimination of either TREK-1 alone or of TREK-1 and TWIK-1 together. We show that neither passive conductance nor *V*_M_ were altered in the double gene knockout mice. Meanwhile, the quantity and expression pattern of other known K^+^ channels were unchanged in TWIK-1^−/−^/TREK-1^−/−^ astrocytes; thus compensatory K^+^ channel expression was unlikely a cause for our functional observations. Our results are inconsistent with the conclusion in a previous study, where virus shRNA delivery was used as gene knockdown approach (Hwang et al., 2014). However, because different experimental approaches are subject to different limitations, such as potential alternation in other gene expression in gene knockout mice and potential off-target effect in shRNA gene knockdown method (Toro Cabrera and Mueller, 2016), caution should be exercised in viewing the discrepancy resulting from different experimental approaches, and a more definite experimental approach would be highly valuable to resolve this inconsistency in the future.

Nevertheless, the function of the highly expressed K_2P_s, TWIK-1 and TREK-1 channels in astrocytes remains to be precisely determined. The present study also calls attention to explore alternative channels contributing to the important but still mysterious passive conductance.

## Author Contributions

YD, WW, BM and MZ conceived the project, YD, CMK, WW, QW, BM and CCA conducted the research. CH contributed TWIK-1 mice study, participated in experimental design and discussion. EEM, RMB created TREK-1 knockout mice and provided consultation to this project. YD and MZ wrote the manuscript. MZ supervised the project. All authors listed, have made substantial, direct and intellectual contribution to the work, and approved it for publication.

## Conflict of Interest Statement

The authors declare that the research was conducted in the absence of any commercial or financial relationships that could be construed as a potential conflict of interest.
